# Significant Progress for Hot-Deformed Nd-Fe-B Magnets: A Review

**DOI:** 10.3390/ma16134789

**Published:** 2023-07-03

**Authors:** Renjie Chen, Xianshuang Xia, Xu Tang, Aru Yan

**Affiliations:** 1 CISRI & NIMTE Joint Innovation Center for Rare Earth Permanent Magnets, Ningbo Institute of Materials Technology and Engineering, Chinese Academy of Sciences, Ningbo 315201, China; chenrj@nimte.ac.cn (R.C.); tangxu@nimte.ac.cn (X.T.); aruyan@nimte.ac.cn (A.Y.); 2 CAS Key Laboratory of Magnetic Materials and Devices, Ningbo Institute of Materials Technology and Engineering, Chinese Academy of Sciences, Ningbo 315201, China; 3 University of Chinese Academy of Sciences, Beijing 100049, China

**Keywords:** Nd-Fe-B, hot-deformed magnet, coercivity, grain boundary diffusion, micromagnetic simulation, magnetization reversal

## Abstract

High-performance Nd-Fe-B-based rare-earth permanent magnets play a crucial role in the application of traction motors equipped in new energy automobiles. In particular, the anisotropic hot-deformed (HD) Nd-Fe-B magnets prepared by the hot-press and hot-deformation process show great potential in achieving high coercivity due to their fine grain sizes of 200–400 nm, which are smaller by more than an order of magnitude compared to the traditional sintered Nd-Fe-B magnets. However, the current available coercivity of HD magnets is not as high as expected according to an empirical correlation between coercivity and grain size, only occupying about 25% of its full potential of the anisotropy field of the Nd_2_Fe_14_B phase. For the sake of achieving high-coercivity HD magnets, two major routes have been developed, namely the grain boundary diffusion process (GBDP) and the dual alloy diffusion process (DADP). In this review, the fundamentals and development of the HD Nd-Fe-B magnets are comprehensively summarized and discussed based on worldwide scientific research. The advances in the GBDP and DADP are investigated and summarized based on the latest progress and results. Additionally, the mechanisms of coercivity enhancement are discussed based on the numerous results of micromagnetic simulations to understand the structure–property relationships of the HD Nd-Fe-B magnets. Lastly, the magnetization reversal behaviors, based on the observation of magneto-optic Kerr effect microscopy, are analyzed to pinpoint the weak regions in the microstructure of the HD Nd-Fe-B magnets.

## 1. Introduction

**Figure 1:**
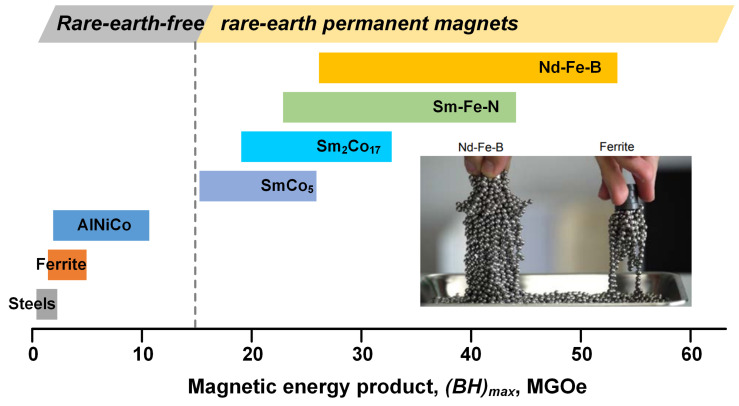
The magnetic energy product ranges for the various families of permanent magnets. The right photograph was first used by K. Hono from National Institute for Materials Science.

Over the past century, there has been significant development in permanent magnetic materials, resulting in a steady increase in their magnetic energy product (*BH*)*_max_* from rare-earth-free permanent magnetic materials ((*BH*)*_max_* < 15 MGOe) to rare-earth permanent magnetic materials ((*BH*)*_max_* ≈ 15–55 MGOe) (Figure 1) [1,2,3,4]. Currently, the Nd-Fe-B based rare-earth permanent magnetic material possesses the highest (*BH*)*_max_* and has been widely applied in numerous industrial fields, such as new energy vehicles and wind power generation [2,3,4,5,6,7]. Among permanent magnetic materials, sintered Nd-Fe-B magnets are highly popular on account of their mature manufacturing technology and excellent magnetic performance, capturing over 50% of the market [8]. However, the high-coercivity sintered magnets depend heavily on the expensive heavy rare-earth elements (HREs) such as dysprosium (Dy) and terbium (Tb) for the application of traction motors working at temperatures above 150 °C [9,10,11,12]. The HREs are used for the substitution of Nd to heighten the anisotropy field (*H_A_*) of the main phase because the *H_A_* values of Dy_2_Fe_14_B (μ_0_*H_A_* = 15.0 T) and Tb_2_Fe_14_B (μ_0_*H_A_* = 22.0 T) are much higher than those of Nd_2_Fe_14_B (μ_0_*H_A_* = 7.6 T). The scarcity of HRE resources has posed a challenge to their sustainability since the “Rare-earth crisis” occurred around 2011. Therefore, numerous researchers are making efforts to develop low-HRE or even HRE-free Nd-Fe-B magnets accompanied with high coercivity [13,14,15]. The application of the grain boundary diffusion process (GBDP) has proven effective in decreasing the usage of HREs in the sintered Nd-Fe-B magnets through the formation of HRE-enriched shells surrounding grains [16,17,18]. Additionally, controlling the microstructure by reducing grain sizes offers another efficient approach to raise coercivity [10,19,20,21,22].

**Figure 2:**
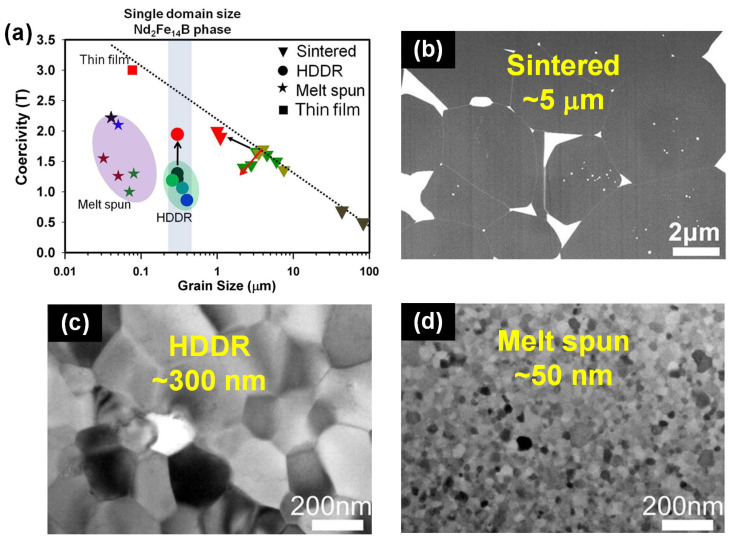
(**a**) The statistical relationship between coercivity and grain size for sintered Nd-Fe-B magnets, HDDR Nd-Fe-B powders and melt-spun Nd-Fe-B ribbons. Typical microstructural features of (**b**) sintered Nd-Fe-B magnets, (**c**) HDDR Nd-Fe-B powders and (**d**) melt-spun Nd-Fe-B ribbons. Reproduced with permission [10]. Copyright 2012, Elsevier.

Figure 2 illustrates the coercivity vs. grain size for different types of Nd-Fe-B magnets, including sintered Nd-Fe-B magnets with a micron level grain size of ~5 μm, hydrogen-disproportionation-desorption-recombination (HDDR) Nd-Fe-B powders with a submicron grain size of ~300 nm and melt-spun Nd-Fe-B ribbons with a nanoscale grain size of ~50 nm [10]. It is clear that as the grain size decreases, the coercivity tends to increase following the equation *H_c_* = a − b ln *D*, where D is the grain size and a and b are the logarithmic fitting coefficients. However, when the grain size decreases below 3 μm, the available coercivity drops significantly due to the oxidation of pulverized fine powders during the conventional powder metallurgy route [23]. Hence, in order to further enhance the coercivity, alternative processing methods must be explored that allow for the reduction in grain sizes to the single domain of the Nd_2_Fe_14_B phase (~250 nm) or even finer, and the oxidation of fine powders can be prevented at the same time. Among various magnet manufacturing processes, the hot-press and hot-deformation process is a promising next-generation industrial process for the production of high-coercivity permanent magnets comprising nanoscale grains. The hot-deformed (HD) Nd-Fe-B magnets, which are produced from melt-spun powders with ultrafine grains (~50 nm), are distinct from the traditional sintered Nd-Fe-B magnets with micron-sized grains. Since the sizes of flake melt-spun powders are much larger than those of grains, the oxidation of nanoscale grains within the flakes are preventable during the process. The HD magnets have grain sizes ranging between 200 and 400 nm and have garnered interest as a promising choice for achieving high coercivity without resorting to HREs.

**Figure 3:**
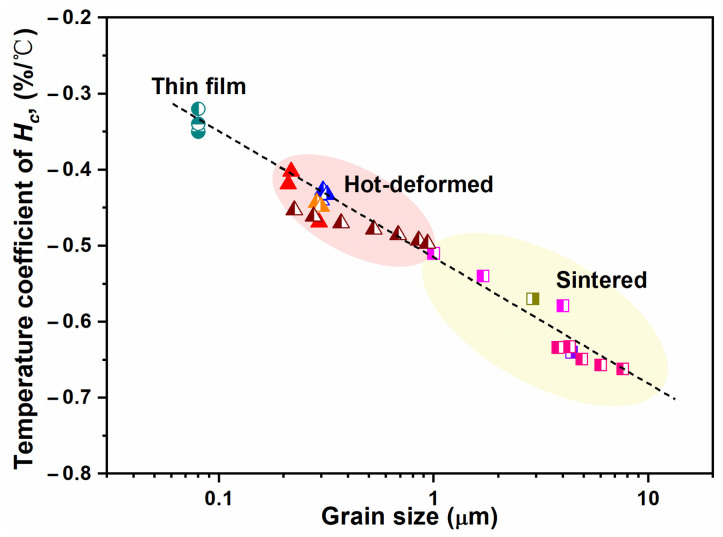
Temperature coefficient of coercivity (25–180 °C) vs. grain size for various types of HRE-free Nd-Fe-B magnets. Reproduced with permission [24]. Copyright 2020, Elsevier.

For the permanent magnets, the thermal stability generally refers to the ability to resist thermal demagnetization at working temperatures (120–200 °C) in various applications. The thermal stability of coercivity is evaluated by the temperature coefficient of coercivity (*β*), which is defined by the following equation:

(1) $$\beta =\frac{H_{c}\left(T\right)-H_{c}(25\;{}^{\circ}C)}{H_{c}\left(25\;{}^{\circ}C\right)\times (T-25)}\;,$$

where *T* is the working temperature of magnets. The relationship between the temperature coefficient of coercivity (25–180 °C) and grain size for various types of Nd-Fe-B magnets (HRE-free) is illustrated in Figure 3 [24]. Similar to the relationship between coercivity and grain size, the temperature coefficient of coercivity also exhibits a logarithmic correlation with grain size. Obviously, compared to the sintered magnets with micron-sized grains, the HD magnets with nanoscale grains show a superior thermal stability of coercivity. Although the HD magnets have great potential in achieving high coercivity both at room temperature and elevated temperatures, the attainable coercivity of HD magnets (1.0–1.5 T) in earlier studies is a far cry from the predicted value considering their ultrafine grain sizes [25,26,27]. This is attributed to the high proportion of ferromagnetic elements such as Fe and Co in grain boundaries, resulting in strong ferromagnetic exchange coupling between hard magnetic Nd_2_Fe_14_B grains [28,29].

**Figure 4:**
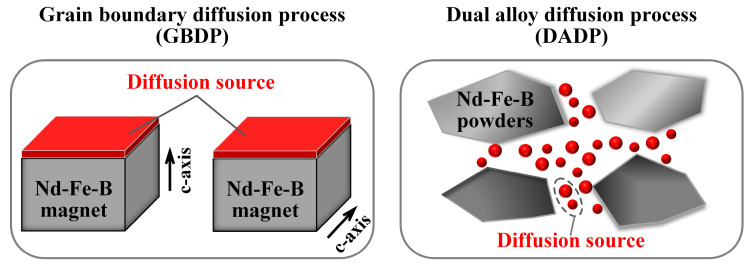
Schematic diagrams of the grain boundary diffusion process and the dual alloy diffusion process.

A lot of research has been carried out to enhance the coercivity of HD magnets with low Dy/Tb or even free Dy/Tb. Thus far, two main methods have been proven effective and greatly developed, namely the grain boundary diffusion process (GBDP) and the dual alloy diffusion process (DADP). Figure 4 depicts the schematic diagrams of the two processes. The GBDP is classified as external diffusion, where the diffusion source is placed at the surfaces of magnets. Owing to the limited diffusion depth, the content of the RE-rich phase near the diffusion surfaces is higher than that inside the magnets. Therefore, the GBDP is usually applied on the lamellar magnets. Unlike GBDP, the DADP is classified as internal diffusion, where the diffusion source is mixed with the melt-spun powders before the cold-pressing and hot-pressing process. Hence, the DADP is preferred for preparing bulk magnets with relatively uniform microstructure and magnetic properties due to the unlimited diffusion depth. Through modifying the microstructure, including the main phase, grain boundary phases, grain size, etc., these two methods have contributed to significantly enhancing the coercivity of HD magnets. On the foundation of the latest progress, this paper presents a comprehensive review of the fundamentals and development of HD Nd-Fe-B magnets. Section 2 summarizes the characteristics of the production process and microstructure of the HD magnets. Section 3 investigates the recent advances regarding the GBDP and DADP in the HD magnets. Section 4 and Section 5 discuss the mechanisms of coercivity enhancement and the magnetization reversal behaviors, respectively, of the HD magnets.

## 2. Production Process and Microstructural Features of Hot-Deformed Magnets

**Figure 5:**
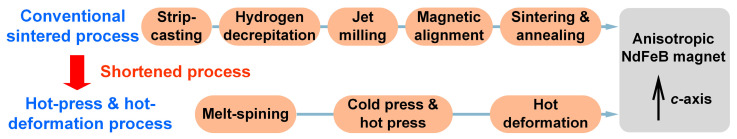
A comparison between the conventional sintered process and hot-press and hot-deformation process for the preparation of anisotropic Nd-Fe-B magnets.

The crystal alignment mechanism employed in the production of HD magnets completely differs from the traditional sintered magnets. In sintered magnets, the crystallographic alignment is achieved via magnetic alignment, while in HD magnets, such alignment is achieved through plastic deformation at high temperatures [30]. Figure 5 shows a comparison between the conventional sintered process and the hot-press and hot-deformation process, which both can produce the anisotropic Nd-Fe-B magnets [31,32]. It is clear that the production process of HD magnets is simpler than that of sintered magnets, showing less processing steps to prepare the anisotropic Nd-Fe-B magnets.

**Figure 6:**
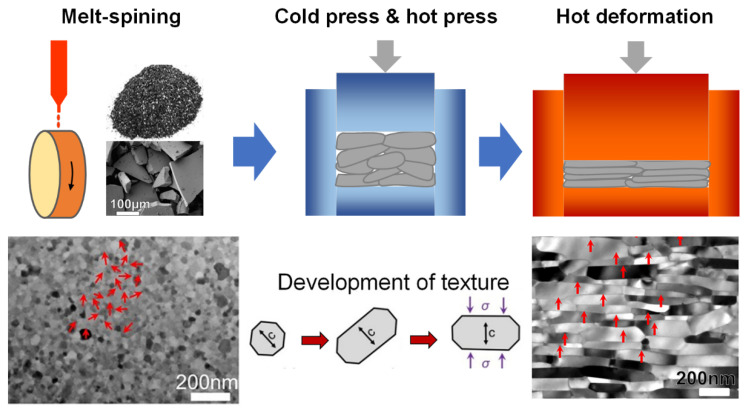
Production process of HD magnets and their microstructural features at different stages. The *c*-axis alignment of grains is indicated by red arrows. Reproduced with permission [41]. Copyright 2018, Elsevier.

**Figure 7:**
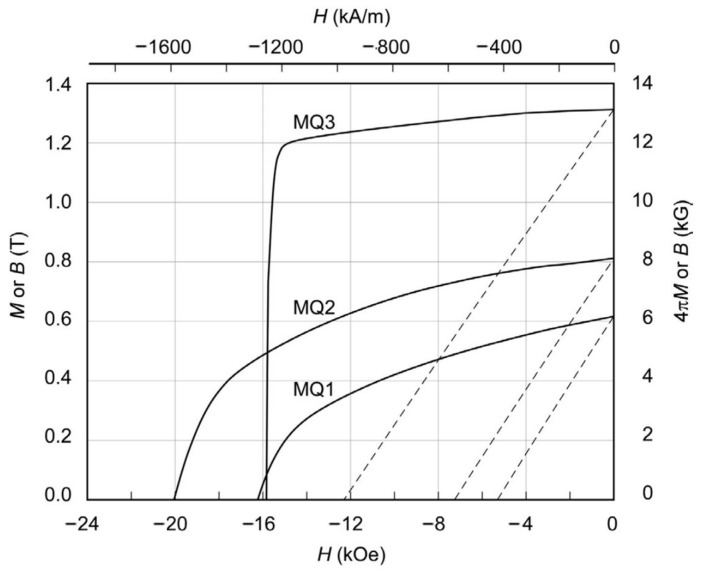
Typical demagnetization curves of MQ1, MQ2 and MQ3 Nd-Fe-B magnets produced from melt-spun powders. Reproduced with permission [42]. Copyright 2022, Elsevier.

The schematic diagrams of producing HD magnets are depicted in Figure 6. The initial step involves preparing the precursors by using a rapid quenching machine, followed by the pulverization of precursors into flaky powders with sizes of 50–300 μm. These powders contain a multitude of Nd_2_Fe_14_B nanocrystalline grains with a random orientation [33,34]. In the second step, the flake powders are cold-pressed at room temperature and then hot-pressed at 650–700 °C to obtain a complete densified isotropic magnet with a grain size of ~50 nm [35,36,37]. The final step involves a hot deformation process, in which the isotropic magnet suffers a plastic flow process at 750–850 °C until a ~70% height reduction, resulting in an anisotropic magnet. The anisotropic HD magnet has highly oriented platelet-shaped grains with sizes of 200–400 nm in diameter and 50–80 nm in thickness. The formation of grain texture in this process involves two aspects, namely the preferential growth of Nd_2_Fe_14_B nanocrystallites along the c-plane and the rotation of Nd_2_Fe_14_B nanocrystallites towards the direction perpendicular to the deformation pressure [35,36,37,38,39,40,41]. The cold-pressed isotropic magnet, hot-pressed isotropic magnet, and HD anisotropic magnet are commonly known as MQ1, MQ2 and MQ3, respectively, with their distinct demagnetization curves illustrated in Figure 7 [42]. Their remanences and (*BH*)*_max_* progressively increase from MQ1 to MQ2 and ultimately to MQ3.

**Figure 8:**
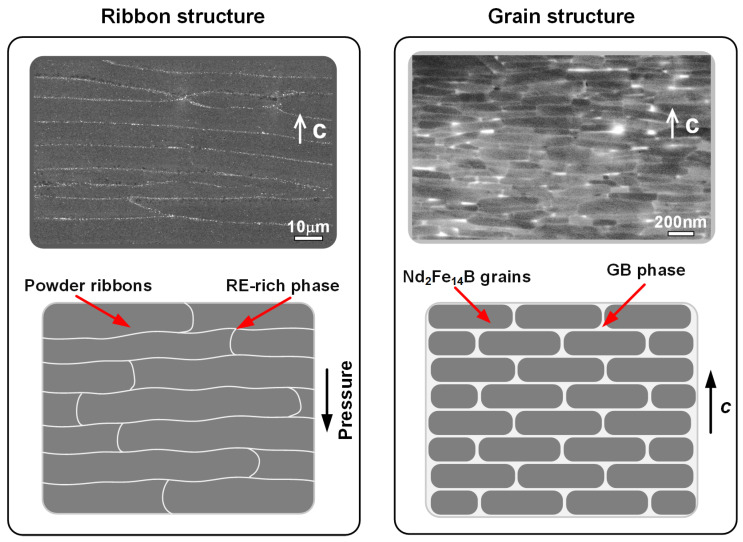
Microstructural features of HD magnets at different scales: ribbon structure at micron scales and grain structure at nanoscales.

Figure 8 illustrates the microstructural features of HD magnets at various scales and provides the corresponding schematic diagrams. At micron scales, the magnets contain a mass of lathy powder ribbons that are stacked perpendicular to the pressure direction. The powder ribbons are separated by the thin RE-rich phases. At the nanoscale, the platelet-shaped grains are highly aligned and enveloped by the intergranular RE-rich phases. The magnetic properties of HD magnets are greatly dependent on their microstructure. For instance, the remanence highly depends on the *c*-axis alignment of grains, while the coercivity is heavily influenced by the intergranular RE-rich phase. For the HRE-free HD magnets, the acquired remanence (1.35–1.50 T) has occupied over 80% of its full potential of the saturation magnetization (μ_0_*M_s_* = 1.61 T) of the Nd_2_Fe_14_B phase, while the acquired coercivity (1.5–2.0 T) is only about 25% of its full potential of the anisotropy field (μ_0_*H_A_* = 7.6 T) of the Nd_2_Fe_14_B phase, indicating that there is still much room for the improvement of coercivity. Therefore, the current research mainly focuses on the development of high-coercivity HD Nd-Fe-B magnets.

**Figure 9:**
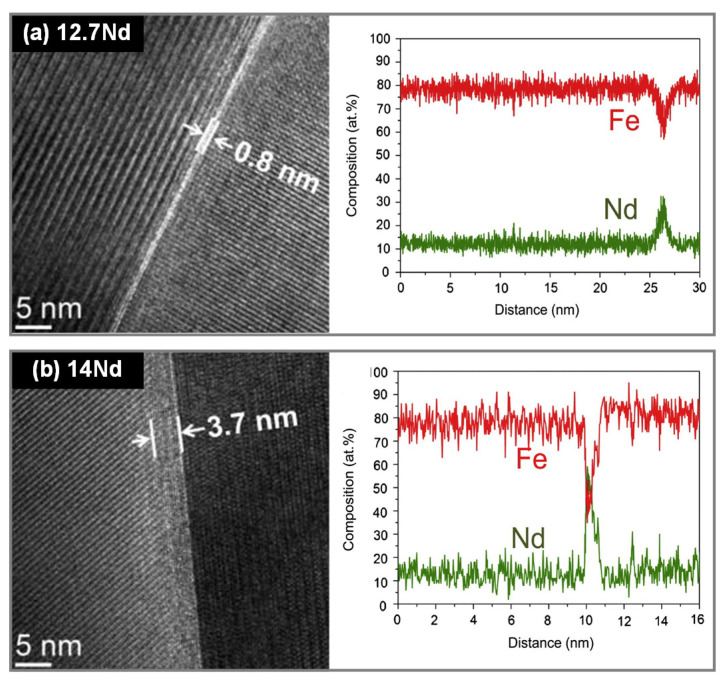
High-resolution transmission electron microscopy (HRTEM) images and compositional profiles across grain boundaries of HD magnets with various Nd contents. Reproduced with permission [45]. Copyright 2013, Elsevier.

The presence of intergranular RE-rich phases is crucial for achieving high coercivity in the HD magnets [43,44]. According to Liu et al. [45], the overall Nd content within the magnets has a great impact on both the thickness and RE content of the grain boundary. Specifically, at an overall Nd content of 12.7 at.%, the grain boundary thickness is approximately 0.8 nm, and the Nd proportion of the grain boundary is roughly 23 at.%, which is believed to be ferromagnetic (Figure 9a). By contrast, at an overall Nd content of 14.0 at.%, the grain boundary thickness increases to approximately 3.7 nm and the Nd proportion of the grain boundary rises to about 46 at.%, which is thought to be nonferromagnetic (Figure 9b). These different features of the grain boundary led to a coercivity of 0.9 T for the 12.7 Nd magnet and 1.8 T for the 14.0 Nd magnet. These results indicate that the thicker and nonferromagnetic grain boundary phase can decouple the hard magnetic grains and enhance the coercivity of magnets.

## 3. Two Routes for High-Coercivity Hot-Deformed Magnets

### 3.1. Progress in Grain Boundary Diffusion Process

**Figure 10:**
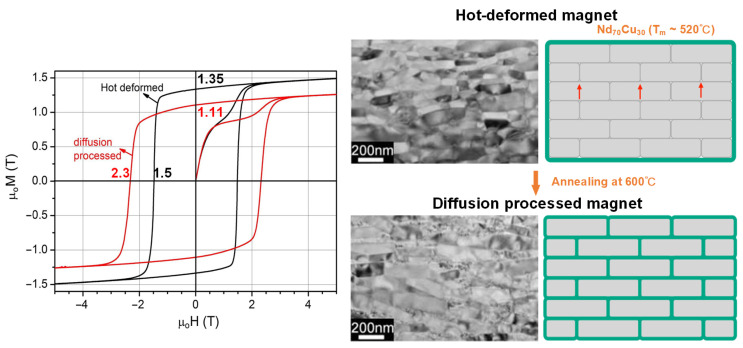
Magnetization curves of the HD magnet and the Nd-Cu infiltrated magnet and their corresponding microstructure. The red arrows in schematic diagram refer to the *c*-axis alignment of grains. Reproduced with permission [29]. Copyright 2013, Elsevier.

**Figure 11:**
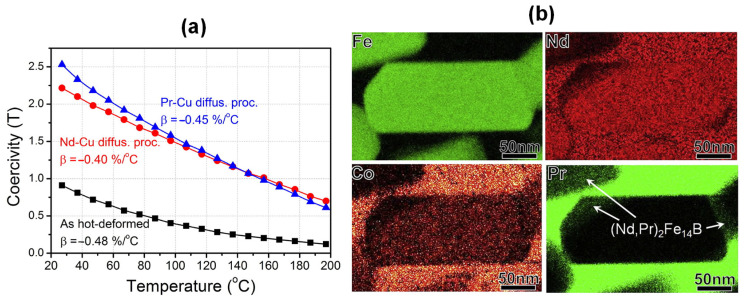
(**a**) Temperature dependence of coercivities for the as-deformed, Nd-Cu infiltrated and Pr-Cu infiltrated magnets, respectively. (**b**) Elemental distribution of Fe, Nd, Co and Pr for the Pr-Cu infiltrated magnet. Reproduced with permission [54]. Copyright 2015, Elsevier.

The GBDP was originally developed for the sintered Nd-Fe-B magnets. In early studies, the GBDP which utilized HRE fluorides [46,47], HRE hydrides [17,48], or HRE alloys [49,50] has been proven successful in improving the coercivity of sintered magnets through the formation of HRE-enriched shells. However, this process requires high temperatures of about 900 °C, making it unsuitable for the nanosized HD magnets due to the significant grain coarsening. Alternatively, low-melting-point HRE-free eutectic alloys such as Nd-Cu [29,51], Nd-Al [52], Pr-Cu [53,54], etc., have been used to infiltrate HD magnets at relatively low temperatures of 500–700 °C, which helps to suppress grain coarsening. Sepehri-Amin et al. [29] reported that a high coercivity of 2.3 T was obtained in a Nd_70_Cu_30_ infiltrated HD Nd-Fe-B magnet (Figure 10). They achieved this by increasing the proportion of RE-rich intergranular phases from 10% to 37% and modifying the thick intergranular phase from ferromagnetic to nonferromagnetic. In subsequent studies, Nd-M (M = Ga, Zn, Al, Mn, etc.) low-melting-point eutectic alloys were attempted as diffusion sources to further enhance the coercivity of HD magnets, and a higher coercivity of 2.5 T was successfully achieved through the diffusion of the Nd_90_Al_10_ alloy [52]. Furthermore, Pr-containing low-melting-point eutectic alloys were also used to increase the coercivity of HD magnets due to the higher *H_A_* of Pr_2_Fe_14_B compared with Nd_2_Fe_14_B. Notably, the Pr_70_Cu_30_ and Pr_90_Cu_10_ diffusion led to the attainment of higher coercivities of 2.56 T and 2.60 T, respectively [53,54]. Unlike Nd-containing eutectic alloys, the diffusion of Pr-containing eutectic alloys not only transformed the intergranular phase into nonferromagnetic but also created the (Nd,Pr)_2_Fe_14_B surface region in the corner of grains (Figure 11), which accounted for the prominent enhancement of coercivity. However, the Pr-Cu infiltrated magnets showed a slight temperature degradation of coercivity in contrast to the Nd-Cu infiltrated magnets, which is due to the faster temperature decline of the *H_A_* of Pr_2_Fe_14_B relative to that of Nd_2_Fe_14_B [54].

**Figure 12:**
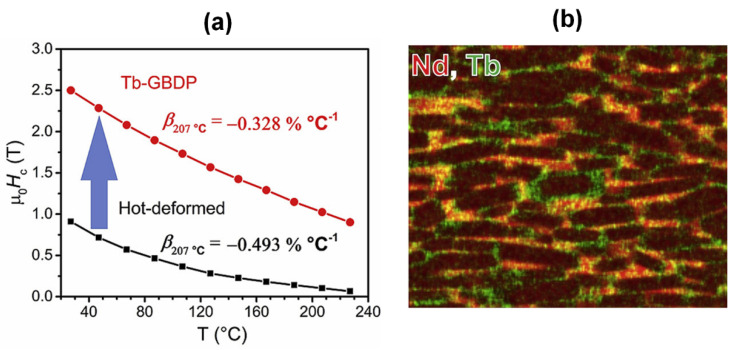
(**a**) Temperature dependence of coercivities for the as-deformed magnet and Nd_60_Tb_20_Cu_20_ infiltrated magnet. (**b**) Typical elemental mapping of Nd and Tb for the Nd_60_Tb_20_Cu_20_ infiltrated magnet. Reproduced with permission [57]. Copyright 2018, Elsevier.

To achieve a superior coercivity and thermal stability of the HD magnets, low-melting-point multielement alloys containing Dy/Tb, such as Nd-Dy-Cu [55], Nd-Dy-Al [56] and Nd-Tb-Cu [57], were utilized in the GBDP. This approach obtained higher coercivities of 2.6 T through the diffusion of Nd_60_Dy_20_Cu_20_ [55] and 2.75 T through the diffusion of Nd_62_Dy_20_Al_18_ [56]. Recently, Li et al. [57] reported that the diffusion of a Tb-containing Nd_60_Tb_20_Cu_20_ alloy achieved remarkable room-temperature magnetic performance with a high coercivity of 2.57 T and a high remanence of 1.38 T, while also delivering outstanding high-temperature magnetic performance with a high coercivity of 1.47 T at 150 °C (Figure 12a). In addition to the exchange decoupling of grains prompted by the nonferromagnetic intergranular phase, the formation of a (Nd,Tb)_2_Fe_14_B shell is another crucial factor contributing to the excellent coercivity and its thermal stability (Figure 12b).

**Figure 13:**
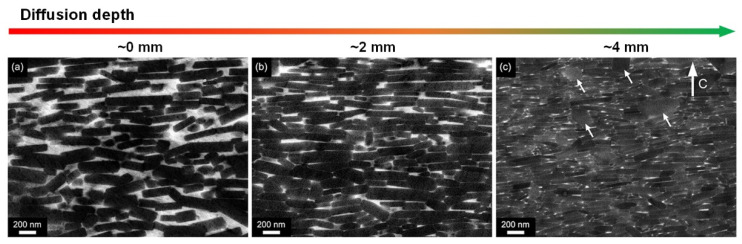
Microstructural features of the infiltrated magnets at different diffusion depths. Reproduced with permission [58]. Copyright 2017, Elsevier.

**Figure 14:**
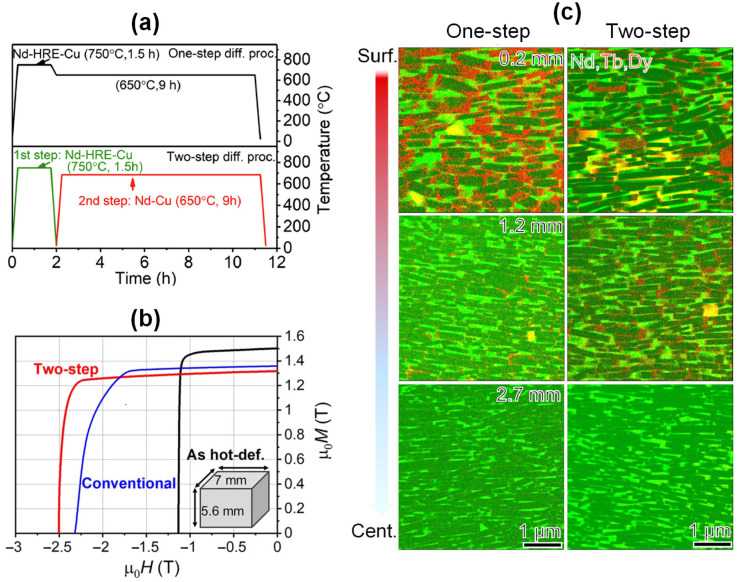
(**a**) Schematic process flows of the one-step diffusion process and two-step diffusion process. (**b**) Typical demagnetization curves of the as-deformed magnets and the diffusion-processed magnets suffered by the conventional diffusion process and the two-step diffusion process. (**c**) Elemental mappings of Nd, Tb and Dy obtained at different depths from the magnet surface of the diffusion-processed magnets suffered by the one-step diffusion process and the two-step diffusion process. Reproduced with permission [59]. Copyright 2021, Elsevier.

On account of the limited diffusion distance, the proportion of the RE-rich phase close to the diffusion surface is much higher than that in the center, and at a diffusion depth of ~4 mm, the infiltrated magnets show a scarce RE-rich phase (Figure 13) [58]. Thus, the HD Nd-Fe-B magnets produced through the GBDP only exhibit both high coercivity and good squareness in lamellar magnets with thicknesses < 4 mm. This greatly restricts the potential application of HD magnets. Significantly, a two-step diffusion process developed by Tang et al. [59] successfully promoted the diffusion depth of HREs. The two-step process involves the diffusion of a high-melting-point Tb_20_Dy_10_Nd_40_Cu_30_ alloy in the first step, followed by the diffusion of a low-melting-point Nd_80_Cu_20_ alloy in the second step (Figure 14a). Thus, both a high coercivity of 2.43 T and an excellent squareness of 0.91 were achieved in 5.6 mm thick HD magnets (Figure 14b). The two-step diffusion process led to a more uniform distribution of the HREs throughout the entire magnet, greatly improving the microstructural uniformity and reducing the gradient of coercivity from the surface to the interior of the thick magnet (Figure 14c).

### 3.2. Progress in Dual Alloy Diffusion Process

**Figure 15:**
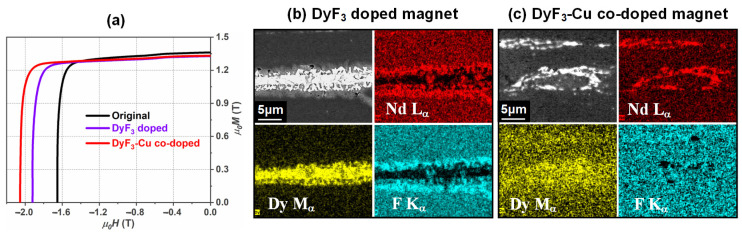
(**a**) Demagnetization curves of the as-deformed, DyF_3_-doped and DyF_3_-Cu-co-doped magnets. SEM-EDS element mappings of Nd, Dy and F at the ribbon interface for the (**b**) DyF_3_-doped magnet and the (**c**) DyF_3_-Cu-co-doped magnet. Reproduced with permission [63]. Copyright 2023, Elsevier.

In early studies, Dy fluoride was investigated as a diffusion source to improve the coercivity of HD magnets through the DADP [60,61,62]. The prevailing hypothesis is that DyF_3_ powders mainly collect at the interface of ribbons and decompose at ~660 °C, followed by Dy diffusion into the interior of powder ribbons during the hot-press and hot-deformation process. However, on account of the high melting point of Dy, the diffusion efficiency of Dy infiltrating into ribbons was limited and most of the Dy aggregated at the ribbon interfaces, resulting in relatively low coercivity levels of 1.6–1.9 T in the 2 wt.% DyF_3_-doped magnets [60,61,62]. Recently, Xia et al. [63] found that the addition of a trace amount of nano-Cu facilitated the infiltration of Dy from the ribbon interfaces into the interior, resulting in higher coercivity levels over 2.0 T (Figure 15). Additionally, binary HRE-containing alloys with lower melting points were attempted to achieve higher coercivity [64,65,66,67,68]. The dual alloy diffusion of 2 wt.% Dy-M (M = Cu, Al, Ga, etc.) resulted in coercivities of 1.7–2.2 T [65,66,67], while 2 wt.% Tb-M (M = Cu, Al, Ga, etc.) achieved higher coercivities of 1.9–2.4 T [65,66,67,68].

**Figure 16:**
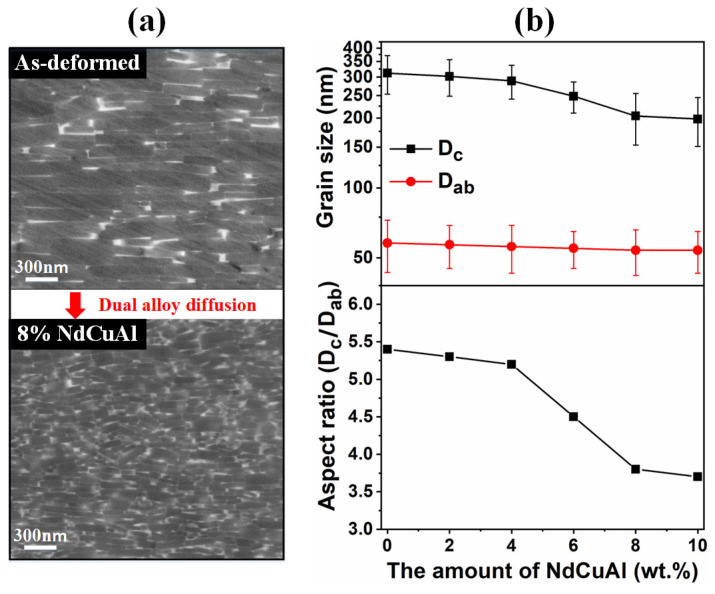
(**a**) Typical grain microstructure of the as-deformed magnets and dual alloy diffused magnets. (**b**) The average grain sizes and the grain aspect ratios vs. the doping content of Nd-Cu-Al alloy powders. Reproduced with permission [69]. Copyright 2020, Elsevier.

Apart from HRE-containing alloys, HRE-free low-melting-point eutectic alloys such as Nd-M and Pr-M (M = Cu, Al, Ga, etc.) were also attempted to enhance the coercivity using the DADP. However, the doping of Nd-M alloy powders, such as Nd-Cu, Nd-Cu-Al and Nd-Fe-Ga-Cu, showed unremarkable results in the increase in coercivity (<2.1 T) [24,65,69,70]. Notably, the doping of Pr-M alloy powders, such as Pr_70_Cu_30_ and Pr_90_Ga_10_, obtained considerable coercivity levels above 2.3 T [71,72]. It is worth mentioning that the grain size and the grain aspect ratio simultaneously decrease with the increase in alloy content (Figure 16) [69]. Therefore, for the DADP without the use of HREs, not only the nonferromagnetic grain boundary phase but also the decreased grain size and grain aspect ratio are responsible for the enhancement of coercivity.

**Figure 17:**
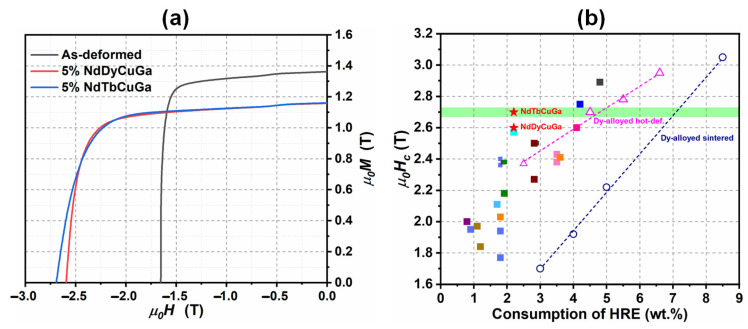
(**a**) Demagnetization curves of the 6 mm thick as-deformed magnet and dual alloy diffused magnets by Nd_35_Dy_35_Cu_15_Ga_15_ and Nd_35_Tb_35_Cu_15_Ga_15_. (**b**) Coercivity vs. the consumption content of HREs for the HRE-diffused and HRE-alloyed HD magnets. Data obtained from refs. [55,56,57,64,65,66,67,68,73,74,75,76,77]. Reproduced with permission [74]. Copyright 2022, Elsevier.

In addition, multicomponent HRE-containing alloys with relatively low melting points were also used in order to improve the utilization efficiency of HREs in the HD magnets. It was reported by Lee et al. [73] that the addition of a Nd_35_Dy_35_Cu_30_ alloy with a low melting point of 610 °C achieved an almost identical coercivity compared to the doping with a Dy_70_Cu_30_ alloy with high melting point of 795 °C, almost doubling the utilization efficiency of the HREs. Recently, Xia et al. [74] also reported that the high coercivity levels of 2.6 T and 2.7 T were obtained in 6 mm thick bulk HD magnets through the addition of Nd_35_Dy_35_Cu_15_Ga_15_ and Nd_35_Tb_35_Cu_15_Ga_15_ alloys with low melting points of 616 °C and 605 °C, respectively, consuming only 2.2 wt.% HREs (Figure 17a). These results are comparable with previous studies that used almost twice the amount of HREs (~4.5 wt.%) (Figure 17b).

**Figure 18:**
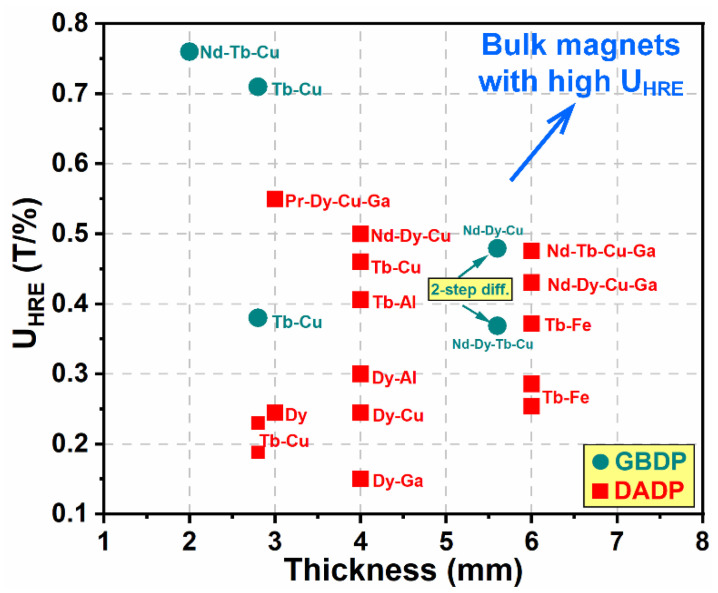
Statistical utilization of HREs vs. the magnet thickness for the HD magnets produced by various preparation processes and diffused by various HRE-containing alloys. Data obtained from refs. [14,57,59,65,66,67,73,74,75,76,77,78]. Reproduced with permission [74]. Copyright 2022, Elsevier.

According to the above review, it can be concluded that the diffusion efficiency of HREs is affected not only by the chemical constituents of HRE-containing alloys, but also by the various diffusion processes (GBDP and DADP). The utilization of HREs is roughly evaluated by the following formula:

(2) $$U_{HRE}=\frac{∆H_{c}}{wt.\%\;of\;HREs}\;,$$

where ∆*H_c_* is the coercivity increment and wt.% of HREs is the mass fraction of HREs consumed in the magnets. Figure 18 provides a comparison between the U_HRE_ and the thickness for the HD magnets produced by various preparation processes and diffused by various HRE-containing alloys. It is evident that the traditional GBDP has high U_HRE_ values for the thin magnets with thicknesses <3 mm, while it shows unsatisfactory outcomes for the thicker magnets. Thus, the two-step diffusion process with an increased process cost is developed for a high U_HRE_ in the thick magnets. The DADP is less effective in achieving high U_HRE_ values for thin magnets (<3 mm) compared to the GBDP, but it can easily realize decent U_HRE_ values in the thick magnets (>3 mm). For the bulk HD magnets with a thickness of 6 mm, the doping of Nd-Dy-Cu-Ga and Nd-Tb-Cu-Ga alloys surpassed that of previous works with regard to achieving a high U_HRE_.

## 4. Coercivity Mechanisms of Hot-Deformed Magnets

Theoretically, if the Nd_2_Fe_14_B grains are perfectly aligned and magnetically isolated from each other, there is potential to achieve a high coercivity of μ_0_*H_A_* = 6.7 T. However, only about 20–40% of such coercivity is achieved among all the Nd-Fe-B-based permanent magnet materials. The following phenomenological equation first proposed by Kronmüller describes the temperature dependence of coercivity (*H_c_*) [79]:

*H_c_*(T) = *αH_A_*(T) − *N_eff_M_s_*(T), (3)

where *H_A_* and *M_s_* refer to the anisotropy field and saturation magnetization of the 2:14:1 phase, respectively, and their values can be obtained from ref. [80]. The first term refers to the influence of the microstructural inhomogeneities depending on the difference in the magnetocrystalline anisotropy between the hard magnetic grains and the microstructural defects, in which *α* is a parameter that relates to the reduction in the *H_A_* caused by the microstructural defects at grain boundaries, grain surfaces and external surfaces of the sample [41,81,82]. The maximum *α* value of 1 is for the magnetically isolated grains without microstructural defects. Conversely, if the microstructural defects completely cover the hard magnetic main phase, the *α* can reach a minimum value of 0. The second term corresponds to the influence of the demagnetization field to the magnetization reversal process, in which *N_eff_* is the effective demagnetization factor that is mainly dependent on the grain size and grain shape [81,82,83]. The *N_eff_* value decreases with the decreased grain size and aspect ratio. Larger *α* and *H_A_* values and smaller *N_eff_* and *M_s_* values imply higher coercivity. The *α* and *N_eff_* values for different types of Nd-Fe-B magnets are listed in Table 1 [10]. There is considerable variation within each type of magnet due to the different conditions from different papers, but it can be concluded that the HD magnets generally exhibit higher *α* and lower *N_eff_*, indicating their greater potential for achieving high coercivities. Table 2 lists the change trend in these magnetic parameters for the HD magnets suffered by different processes. Both the GBDP and DADP increase *α* through modifying the microstructural defects of grains and the GB. For the HRE-containing alloys, the diffusion of them increases *H_A_* and decreases *M_s_* because the HREs diffuse into a 2:14:1 phase. Notably, grain growth is almost inevitable during the GBDP, while it is inhibited during the DADP [69,72]. Therefore, the change in *N_eff_* exhibits the opposite trend between the GBDP and DADP.

**Figure 19:**
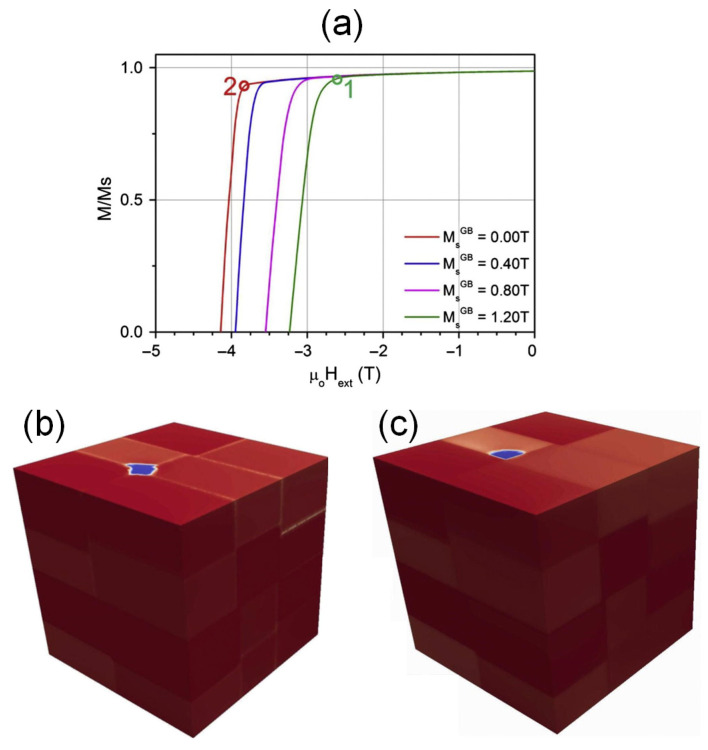
(**a**) Simulated demagnetization curves of the models for HD magnets in which Nd_2_Fe_14_B grains are separated by a 4 nm thick grain boundary phase. The μ_0_*M_s_* of the grain boundary phase varies from 0.0 T to 1.2 T. The snapshots of magnetization reversal process for the models with grain boundary μ_0_*M_s_* of (**b**) 1.2 T and (**c**) 0.0 T at nucleation fields. Reproduced with permission [45]. Copyright 2013, Elsevier.

A finite element micromagnetic simulation is a highly practical tool for studying the impact of microstructure, such as grain boundary phase composition, grain size, grain shape, etc., on the magnetization reversals and coercivity mechanisms of Nd-Fe-B permanent magnets [84]. The influence of the grain boundary phase on the coercivity of HD magnets was simulated by Liu et al. [45]. Figure 19a presents the simulated demagnetization curves of the modeled HD magnets with Nd_2_Fe_14_B grains separated by a 4 nm thick grain boundary phase. By varying the μ_0_*M_s_* and exchange stiffness (*A*) of the grain boundary phase decreased from 1.2 T to 0.0 T and from 8 pJ/m to 0 pJ/m, respectively, the coercivity and nucleation field were enhanced. The decreased ferromagnetism of the grain boundary phase played a critical role in this enhancement. Figure 19b,c showcase snapshots of the magnetization configurations at the nucleation fields for the models with ferromagnetic and nonferromagnetic grain boundary phases, respectively. The presence of a nonferromagnetic phase at the grain boundary effectively inhibits the transmission of a reversed magnetic domain to the surrounding grains. Hence, the pinning strength of the grain boundary phase against the motion of domain walls is a function of the magnetism of the grain boundary phase.

**Figure 20:**
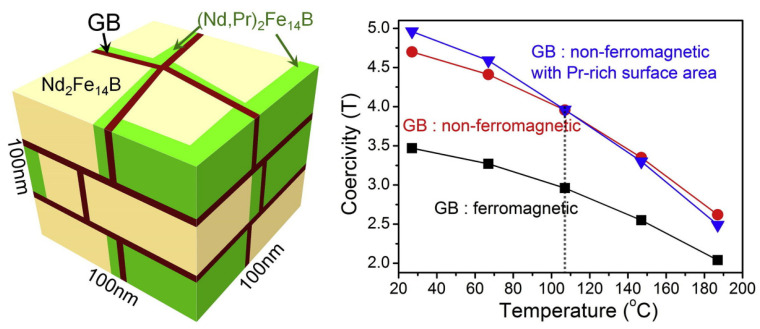
Modeled HD Nd-Fe-B magnet and the simulated coercivity vs. temperature of HD magnet with three different models. Reproduced with permission [54]. Copyright 2015, Elsevier.

It was described above that the Pr-Cu infiltration resulted in a greater increase in coercivity at room temperature, while it showed an inferior thermal stability of coercivity in comparison to the Nd-Cu infiltration [54]. This was likely due to the presence of a (Nd,Pr)_2_Fe_14_B shell. A micromagnetic simulation was utilized to investigate how the presence of the (Nd,Pr)_2_Fe_14_B shell affected the temperature dependence of coercivity, as shown in Figure 20. The results indicated that the coercivity of the model with the (Nd_0_._5_Pr_0_._5_)_2_Fe_14_B shell remained higher than that without until the temperature reached 107 °C. The modeled sample containing the (Nd_0_._5_Pr_0_._5_)_2_Fe_14_B phase exhibited a sharper decrease in coercivity with rising temperature, suggesting that partial Pr substitution for Nd on the surface of platelet-shaped grains slightly worsened the thermal stability of coercivity.

**Figure 21:**
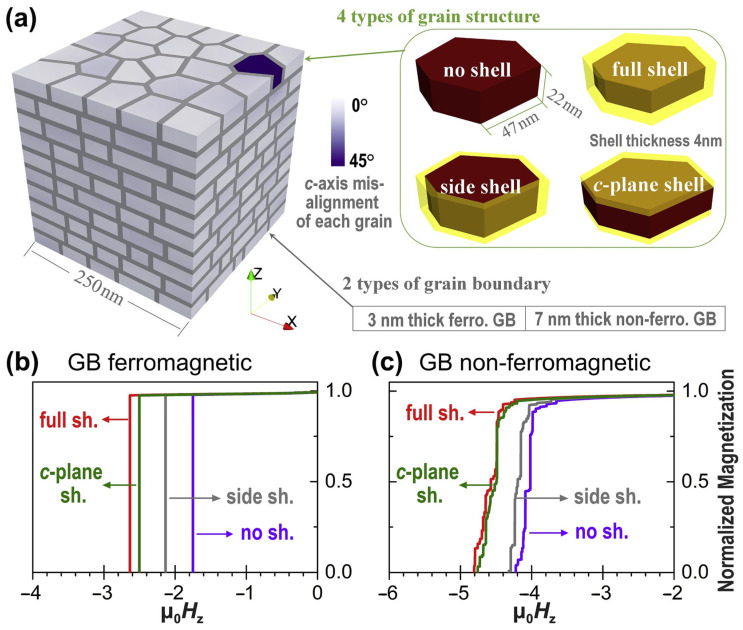
(**a**) Micromagnetic models of the HD Nd-Fe-B magnets with four configurations of grain structure. (**b**,**c**) Simulated demagnetization curves of the various models with different grain structures. The grain boundary phase is assumed to be ferromagnetic in (**b**) and nonferromagnetic in (**c**). Reproduced with permission [57]. Copyright 2018, Elsevier.

It was observed that the HRE-enriched shells on the edge of platelet-shaped Nd_2_Fe_14_B grains were incomplete after the GBDP of HRE-containing alloys in previous works [56,57]. To better understand the contribution of HRE-enriched shells at different surfaces of Nd_2_Fe_14_B grains to magnetization reversals and coercivity, micromagnetic simulations were implemented. As shown in Figure 21a, the HD samples were modeled utilizing four unique configurations: (i) Nd_2_Fe_14_B grains with no shell, (ii) Nd_2_Fe_14_B cores encompassed by entirely Tb-rich shells, (iii) solely the side of Nd_2_Fe_14_B grains covered by a Tb-rich shell and (iv) the c-plane surface of Nd_2_Fe_14_B grains covered by a Tb-rich shell [57]. In the exchange-coupled samples, the formation of Tb-rich shells on the side of the Nd_2_Fe_14_B grains resulted in a coercivity increase from 1.8 T to 2.2 T (Figure 21b). When the Tb-rich shells were located at the c-plane of Nd_2_Fe_14_B grains, a higher coercivity of 2.5 T was obtained. Additionally, the highest coercivity of 2.6 T was achieved when the Tb-rich shells fully covered the Nd_2_Fe_14_B grains. In the exchange-decoupled samples (Figure 21c), the coercivity only slightly enhanced from 4.2 T to 4.3 T when the Tb-rich shells occurred at the side of grains. A similar coercivity of 4.8 T was noted when the Tb-rich shells were formed at the c-plane of the Nd_2_Fe_14_B grains or when they entirely coated the grains. It is noteworthy that for both exchange-coupled and exchange-decoupled models, the c-plane Tb-rich shells played a more significant role in enhancing the coercivity than when the Tb-rich shells were at the side.

**Figure 22:**
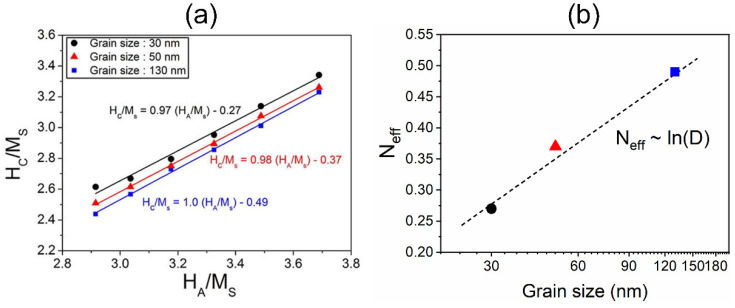
(**a**) *H_c_*/*M_s_* vs. *H_A_*/*M_s_* of the models with various average grain sizes. The parameters of *α* and *N_eff_* are obtained by a linear fitting of Equation (3). (**b**) *N_eff_* values vs. grain size of the micromagnetic models. Reproduced with permission [85]. Copyright 2014, Elsevier.

**Figure 23:**
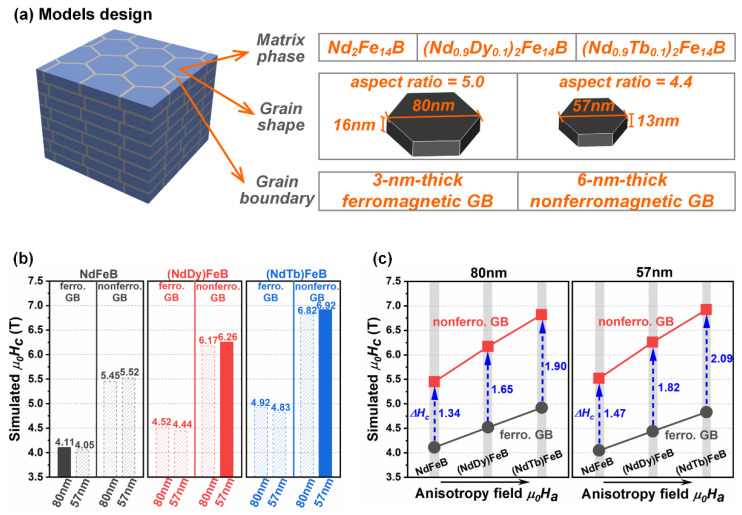
(**a**) Micromagnetic models designed by different structures. (**b**) Comparison of the simulated coercivity values of Nd-Fe-B models with different 2:14:1 phases, grain sizes and grain boundary phases. (**c**) Simulated coercivity value dependence on the *H_A_* of 2:14:1 phases for the models with various grain sizes and grain boundary phases. Reproduced with permission [74]. Copyright 2022, Elsevier.

The results of micromagnetic simulations in a previous work [85] revealed that the coercivity of exchange-coupled Nd-Fe-B magnets can be enhanced by decreasing the grain size due to the smaller effective demagnetization constant, *N_eff_*, as demonstrated in Figure 22. It is worth mentioning that, for the GBDP, the impact of grain size on the coercivity is usually not considered in the micromagnetic simulations because the grain growth is slight. However, the decreases in the grain size and the grain aspect ratio during the DADP are significant and cannot be ignored. Therefore, comprehensive simulations were conducted to reveal the importance of various factors, including the composition of the 2:14:1 phase, the grain size, the grain aspect ratio and the nature of the grain boundary phase, with respect to the enhancement in coercivity [74]. As depicted in Figure 23a, the design of the models involved three compositions of 2:14:1 phases (Nd_2_Fe_14_B, (Nd_0_._9_Dy_0_._1_)_2_Fe_14_B and (Nd_0_._9_Tb_0_._1_)_2_Fe_14_B), two grain shapes (80 × 16 nm^2^ with an aspect ratio of 5.0 and 57 × 13 nm^2^ with an aspect ratio of 4.4), and two grain boundary phases (a 3 nm thick ferromagnetic grain boundary phase and a 6 nm thick nonferromagnetic grain boundary phase). As seen in Figure 23b, it is evident that the coercivity is weakly influenced by the grain size while strongly affected by the composition of the 2:14:1 phase and the nature of the grain boundary phase. This indicates that the composition of the 2:14:1 phase and nature of the grain boundary phase play a more crucial role than the grain size in enhancing the coercivity. Figure 23c plots the simulated coercivity values vs. the *H_A_* of the 2:14:1 phases for models with different grain sizes and grain boundary phases. The models with nonferromagnetic grain boundary phases exhibit higher coercivity values in comparison to those with ferromagnetic grain boundary phases. Additionally, the coercivity increment caused by the nonferromagnetic grain boundary phases demonstrates a positive relationship with the *H_A_* of the 2:14:1 phase, while exhibiting negative relations with the grain size and grain aspect ratio. These results indicate that a higher *H_A_* of the 2:14:1 phase, coupled with smaller grain sizes and grain aspect ratios, increases the positive effect of the nonferromagnetic grain boundary phase in enhancing coercivity.

## 5. Magnetization Reversal Behaviors of Hot-Deformed Magnets

**Figure 24:**
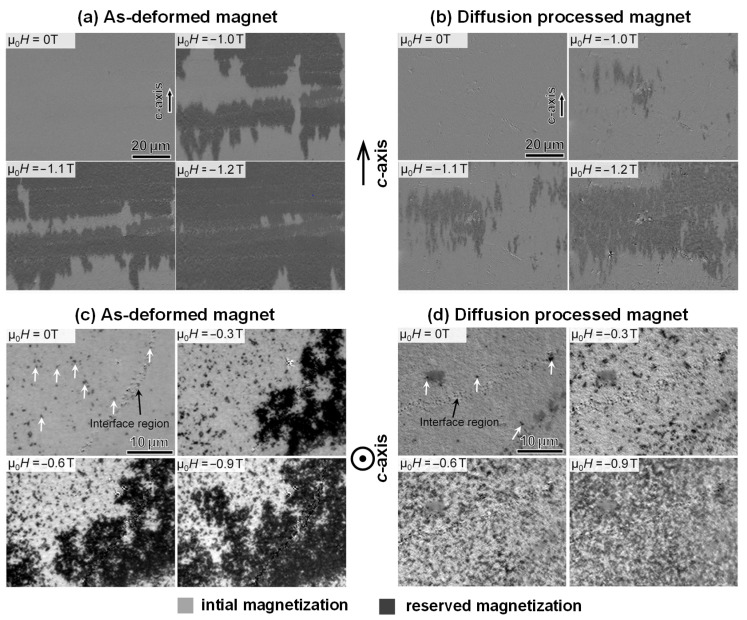
The magnetization reversal process of the (**a**) as-deformed magnet and (**b**) diffusion-processed magnet at 75 °C. The *c*-axis is in-plane. The magnetization reversal process of the (**c**) as-deformed magnet and (**d**) diffusion-processed magnet. The *c*-axis is out-of-plane. Reproduced with permission [86]. Copyright 2022, Elsevier.

Understanding the evolution of magnetization reversal in HD Nd-Fe-B magnets before and after the GBDP is crucial for the strategy of coercivity enhancement. Figure 24a,b illustrate the magnetization reversal processes of the as-deformed and diffusion-processed magnets, respectively, with the *c*-axis in-plane, observed by magneto-optic Kerr effect (MOKE) microscopy [86]. For the as-deformed magnet, cascade propagation of domain walls was observed in the lateral direction perpendicular to the *c*-axis when the applied magnetic field reached −1.0 T. Conversely, for the diffusion-processed magnet, the propagation of reversed domains occurs along the lateral direction without the cascade propagation, and a higher external field is needed to drive the reversal of domains. The change processes of the magnetization reversal of the as-deformed and diffusion-processed magnets with the *c*-axis out-of-plane were also observed, as shown in Figure 24c,d. The cascade-like propagation of domain reversals that exists in the as-deformed magnet transforms into the magnetization reversals in the individual grains of the diffusion-processed magnet. The reason for this transition is the strengthening of the domain wall pinning force when intergranular phases shift from ferromagnetic to nonferromagnetic after the GBDP.

**Figure 25:**
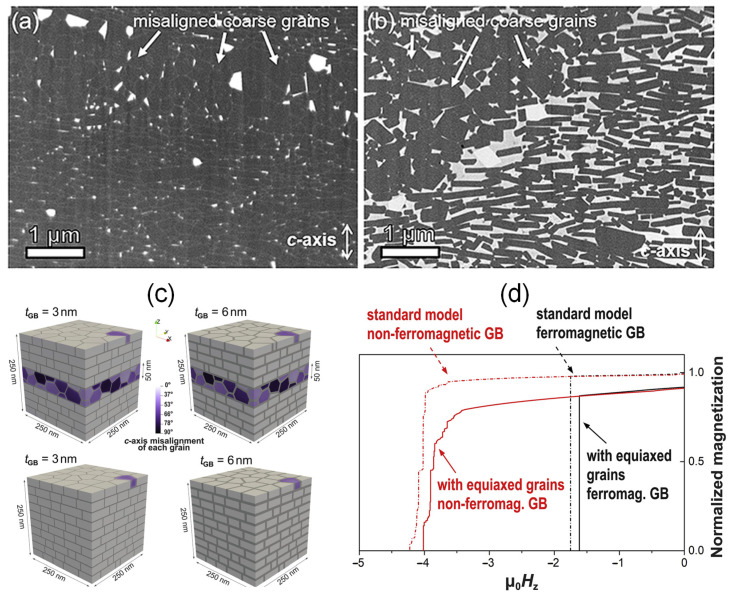
Regions with misaligned CGs in the (**a**) as-deformed magnet and (**b**) diffusion-processed magnet. (**c**) Four models of HD magnets with and without misaligned CGS for 3 nm thick ferromagnetic and 6 nm thick nonferromagnetic grain boundary phases. (**d**) Simulated demagnetization curves of the coupled and decoupled models with and without misaligned CGs. Reproduced with permission [86]. Copyright 2022, Elsevier.

It is worth noting that the reversed magnetic domains originated near the interfaces of the melt-spun ribbons (Figure 24c), which are the weak regions in the microstructure of HD magnets leading to the low coercivity of magnets. Typically, the strongly misaligned equiaxed coarse grains (CGs) are distributed at the ribbon interfaces, as shown in Figure 25a,b. To investigate the effect of equiaxed CGs on coercivity, micromagnetic simulations were performed. Figure 25c illustrates four models of HD Nd-Fe-B magnets with and without misaligned CGs for a thin (3 nm) ferromagnetic grain boundary phase and a thick (6 nm) nonferromagnetic grain boundary phase, and Figure 25d shows their simulated demagnetization curves. For the exchange-coupled models with a thin ferromagnetic grain boundary phase, a relatively low coercivity of 1.62 T was obtained in this model including isotropic equiaxed CGs. However, by simply removing these CGs, a higher coercivity of 1.75 T was achieved. For the exchange-decoupled models with a thick nonferromagnetic grain boundary phase, the model with isotropic equiaxed CGs had a coercivity 4.0 T. The highest coercivity of 4.2 T was achieved when the model was free of isotropic equiaxed CGs. It should be noted that the inclusion of isotropic equiaxed grains substantially reduces the remanence of the samples.

**Figure 26:**
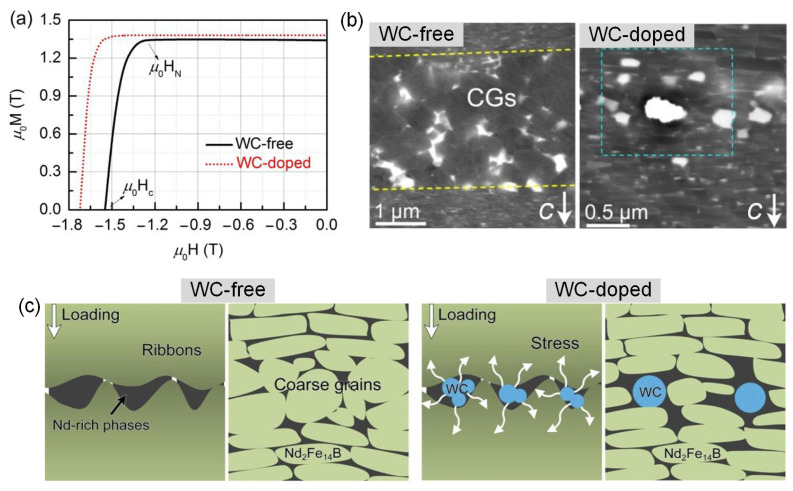
(**a**) Demagnetization curves of WC-free and WC-doped HD magnets. (**b**) CG region between the powder ribbons in the WC-free and WC-doped HD magnets. The CG region is marked by dotted lines. The *c*-axis is in-plane. (**c**) Schematic diagrams of the local stress reinforcement influenced by WC. Reproduced with permission [89]. Copyright 2019, Elsevier.

Therefore, inhibiting the misaligned CGs at the ribbon interfaces is feasible to achieve a higher coercivity of HD magnets [87]. Reports by Zheng et al. [88] and Wang et al. [89] indicated that the doping of 1.0 wt.% high-melting-point WC nano-particles can enhance the coercivity of HD magnets, and their demagnetization curves are shown in Figure 26a. The microstructure observation revealed that the WC nano-particles mainly existed at the ribbon interfaces. On account of the hard WC nano-particles inducing local compressive stress, the excessive growth of Nd_2_Fe_14_B grains at the ribbon interfaces is effectively inhibited, reducing the width of the CG region (Figure 26b). Figure 26c is depicted to explain the CG suppression mechanism. When the interfaces are free of WC, the liquid RE-rich phases aggregate at the interspaces between ribbons and alleviate the local stress loaded on the neighboring grains, resulting in the fast formation of CGs. Conversely, the imported WC dopants increase the local effective stress, adjusting the anisotropic growth of 2:14:1 phases and promoting the *c*-axis alignment of grains.

**Figure 27:**
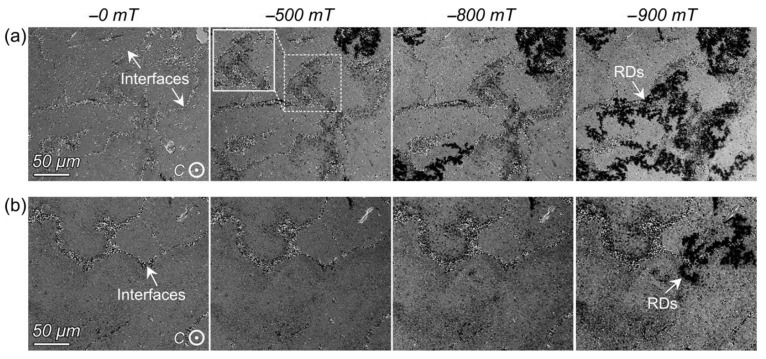
Dynamic magnetization reversal processes of the (**a**) WC-free magnet and (**b**) the WC-doped magnets. The spotty reversed domains are indicated by a frame in (**a**). The direction of applied magnetic field is out-of-plane. Reproduced with permission [89]. Copyright 2019, Elsevier.

To reveal the influence of the WC addition on the microstructure and magnetic properties, the magnetization reversal processes of the WC-free and WC-doped HD magnets were observed by MOKE microscopy (Figure 27). It is clear that the magnetization reversal required a higher negative field for the WC-doped sample compared to the WC-free sample containing the CG structure. In the WC-free sample, the spotty reversed domains (RDs) firstly appeared at the interfaces of ribbons (indicated by a frame in Figure 27a), identified as the regions containing non-oriented CGs. With the increase in the external magnetic field from 500 mT to 900 mT, these spotty domains transformed into coarser dendritic domains and eventually propagated into the interior of ribbons. In the WC-doped sample with reduced CG regions, the magnetization reversal was remarkably hindered, and a few dendritic RDs were seen at the interfaces of ribbons until the negative field increased to 900 mT. These results indicated that the reduction in the CG structure decreased the low-field nucleation probability for magnetization reversal, contributing to coercivity enhancement.

## 6. Strategy for Higher Magnetic Properties of Hot-Deformed Magnets

**Figure 28:**
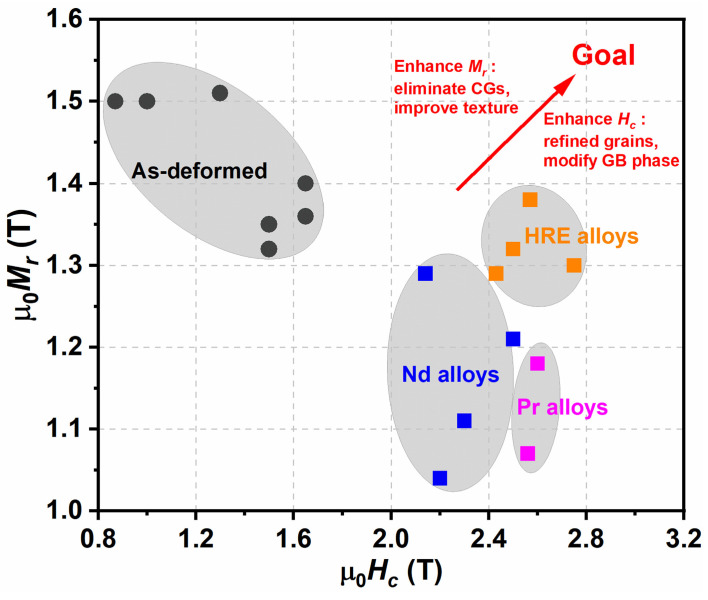
Map of magnetic properties for the as-deformed magnets and diffusion-processed magnets by different types of alloys. Data obtained from refs. [14,29,52,53,54,56,57,59,82].

To completely meet the requirements for the traction motors of (hybrid) electric vehicles at working temperature ranges of 20–180 °C, the magnetic properties of permanent magnets including μ_0_*H_c_* > 2.5 T, μ_0_*M_r_* > 1.35 T and μ_0_*H_c_* @180 °C > 0.8 T should be achieved [41]. Figure 28 shows a map of magnetic properties for the as-deformed magnets and diffusion-processed magnets by different types of alloys. It is clear that the HD magnets diffused by HRE-containing multielement alloys show superior magnetic properties and basically meet the needs of traction motors. According to the current understanding, the further enhancement of the magnetic properties for the HD magnets with lower HREs should modify their microstructure in the following aspects: (i) ultrafine grains and excellent grain alignment, (ii) eliminated CGs, (iii) uniform thin nonferromagnetic grain boundary phase and (iv) fully covered HRE-rich shells. For the HD magnets without HREs, the ideal microstructure and targeted magnetic properties cannot be easily realized only relying on the current GBDP and DADP, and the optimized diffusion processes or other new approaches should be explored for the wider application prospect of the HD Nd-Fe-B magnets.

## 7. Summary

Anisotropic HD Nd-Fe-B magnets offer a promising option for developing high-coercivity magnets due to their nanoscale grains. To further enhance the coercivity and its thermal stability of HD magnets, two effective approaches were developed: the grain boundary diffusion process and the dual alloy diffusion process. These processes modify the microstructure of the magnets, including the composition of the 2:14:1 phase, the nature of intergranular RE-rich phases, grain sizes, etc., without requiring or with low levels of heavy rare-earth elements. In this context, the mechanisms of coercivity enhancement of the HD magnets processed by the grain boundary diffusion process or the dual alloy diffusion process are summarized and discussed based on the numerous results of microstructure observations and micromagnetic simulations, revealing the respective role of the main phase, intergranular phases and grain size on the increased coercivity. Furthermore, the magnetization reversal behaviors of the as-deformed magnets and the diffusion-processed magnets are observed and discussed by directly observing the migration of magnetic domains. The exchange-coupled as-deformed magnets tend to exhibit cascade-like magnetization reversal, and the reversal process often starts in the regions of misaligned CGs. In contrast, magnetization reversal in the exchange-decoupled diffusion-processed magnets also starts at misaligned grains, but it is hindered by nonferromagnetic grain boundary phases, significantly reducing the cascaded propagation of domain walls. Thus, removing weak regions in the microstructure, such as misaligned CGs, is a practical solution to optimize the microstructure and magnetic properties of HD magnets.

## Figures and Tables

**Table 1:** The *α* and *N_eff_* values for various types of Nd-Fe-B magnets. Reproduced with permission [10]. Copyright 2012, Elsevier.

Magnet Types	*α*	*N_eff_*
Sintered magnets	0.6–0.7	1.4–1.8
Nanocrystalline magnets	0.6–0.9	0.75–1.0
HD magnets	~0.8	0.8–1.4
Thin film magnets	0.26–0.41	0.05–0.42

**Table 2:** Change trend of magnetic parameters influenced by the different diffusion processes. The “↑” refers to the up-trend and “↓” refers to the downtrend.

Diffusion Process	Diffusion Source	*α*	*H_A_*	*N_eff_*	*M_s_*
GBDP	HRE-free alloys	↑		↑	
	HRE-containing alloys	↑	↑	↑	↓
DADP	HRE-free alloys	↑		↓	
	HRE-containing alloys	↑	↑	↓	↓

## Data Availability

No new data were created in this study.
